# The diagnostic performance evaluation of Panbio and STANDARD Q coronavirus disease 2019 antigen tests against real-time polymerase chain reaction in southern Ethiopia

**DOI:** 10.1038/s41598-024-55309-w

**Published:** 2024-02-24

**Authors:** Elias Tamene, Alemitu Beyene, Hanibale Atsbeha, Techalew Shimelis

**Affiliations:** School of Medical Laboratory Science, Hawassa University, P.O.Box: 1560, Hawassa, Ethiopia

**Keywords:** Rapid diagnostic test, COVID-19, RT-PCR, SARS-CoV-2, Sensitivity, Specificity, Microbiology, Medical research

## Abstract

The coronavirus disease 2019 (COVID-19) pandemic has created a public health crisis**.** This study aimed to evaluate the diagnostic performance of the Panbio and STANDARD Q COVID-19 antigen rapid diagnostic tests (RDTs) against the real-time polymerase chain reaction (RT-PCR) at one of the largest hospitals in southern Ethiopia. Nasopharyngeal samples, which were collected during the pandemic from individuals suspected of COVID-19 and stored at − 70 °C, were analyzed in June and July 2022. The performance of the Panbio COVID-19 antigen tests was evaluated in 200 randomly selected nasopharyngeal samples (100 positives and 100 negatives for severe acute respiratory syndrome 2 by RT-PCR). The STANDARD Q test was evaluated using 100 positive and 50 negative samples. The respective sensitivity, specificity, positive predictive value and negative predictive values were 88%, 99%, 98.9% and 89.2% for the Panbio test and 91%, 98%, 98.9% and 84.5%, for the STANDARD Q test. The kappa values were 0.87 for the Panbio and 0.86 for the STANDARD Q test. Based on the findings presented here, the RDTs could be utilized as an alternative to conventional RT-PCR when it is challenging to diagnose COVID-19 owing to a lack of time, skilled lab personnel, or suitable equipment or electricity.

## Introduction

Since it was initially discovered in late December 2019 in Wuhan City, China, the SARS-CoV-2, which is the cause of the COVID-19 pandemic, has become a global health issue^1^. The SARS-CoV-2 testing capacity has been regarded as a fundamental factor in achieving pandemic control around the globe^2^. Thus, ensuring an early and accurate diagnosis of the viral infection and sufficient quarantine measures for those infected was considered a key aspect of controlling viral transmission^3^.

The gold standard for the diagnosis of SARS-CoV-2 infection is the RT-PCR assay^4^. As the entire procedure can be completed in a closed tube, RT-PCR is significantly quicker than other available virus isolation methods and has a lower risk of contamination or errors. The test is still the most accurate method with promising sensitivity and specificity available for the detection of SARS-CoV-2 infection^5^. However, the RT-PCR assay heavily depends on skilled personnel, expensive equipment, and advanced labs with adequate power supplies. Additionally, sample analysis using this method typically requires 4–6 h, not including the time for transportation to laboratories, which lengthens the turnaround time. Consequently, RT-PCR techniques are challenging to implement in countries with limited resources and have a limited capacity to track SARS-CoV-2 transmission on a pandemic scale^6^.

In order to overcome such limitations, several RDTs were introduced to the market and are currently available in clinical practice for the detection of the SARS-CoV-2 virus. Among these, the two commonly used assays in Ethiopia are the Panbio and the STANDARD Q COVID-19 RDTs, both employing a lateral flow assay in a cassette-based format with a visual read-out^7^. These RDTs are simple to use and interpret, do not require highly skilled personnel or a laboratory setup, and yield results in less than 30 min, all of which reduce procedure costs and strengthen public health responses^8^. The necessity of thorough evaluations across several geographic locations must be emphasized since the test performance for such products is impacted by variables like investigated population and genetic diversity of causative organisms. However, limited evidence exists regarding the diagnostic usefulness of this product in Ethiopian contexts. Therefore, this study aimed to evaluate the diagnostic performance of Panbio and STANDARD Q COVID-19 RDTs against the RT-PCR from stored samples in southern Ethiopia.

## Methods

### Study setting and samples

**Figure 1:**
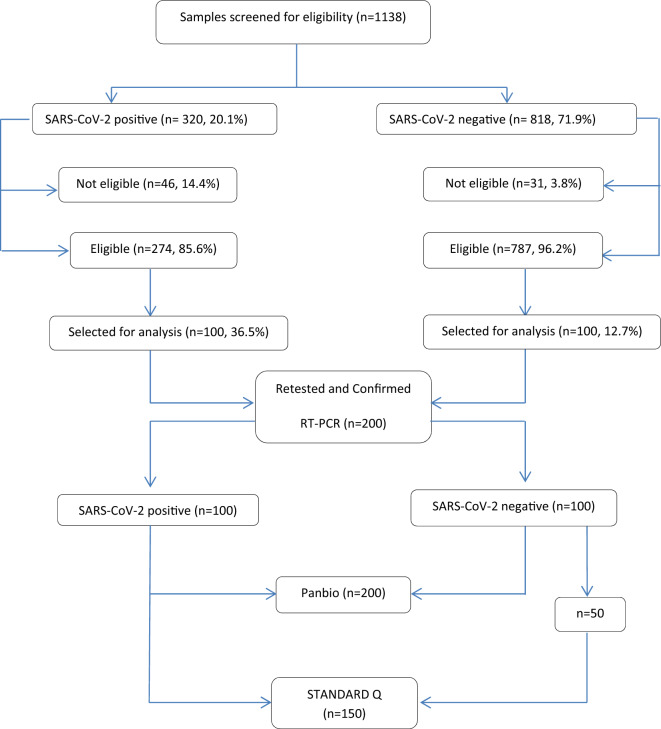
Flow chart of enrolment of the study participants.

The study was conducted at the COVID-19 Testing Centre of Hawassa University Comprehensive Specialized Hospital (HUCSH) in Hawassa City, southern Ethiopia. During the pandemic, there was one quarantine, two isolation, and two treatment centres in Hawassa. The COVID-19 treatment centre at HUCSH is one of the first facilities in the country to offer care services to patients with confirmed COVID-19^9^. It has 100 beds total, including six beds in the intensive care unit, four of which have mechanical ventilation. Trained staff in the hospital have been working to reduce the impacts of the COVID-19 pandemic since the outbreak began in Ethiopia by raising community awareness creation, providing quarantine and treatment services, and conducting COVID-19 research projects. Individuals suspected of COVID-19 were routinely screened for SARS-CoV-2 using RT-PCR. A 5% of the SARS-CoV-2 negative and all positive nasopharyngeal samples were stored at − 70 °C for research purposes. Nasopharyngeal samples, which were collected and stored after being tested for SARS-COV-2 from January 10 to May 30, 2022, were considered for the current analysis. The performance of the Panbio COVID-19 antigen tests was evaluated from June 1 to July 30, 2022, in 200 randomly selected nasopharyngeal samples (100 positives and 100 negatives for SARS-CoV-2) by lottery method. The STANDARD Q test was evaluated using 100 positive samples; however, due to the shortage of test kits, we only tested 50 negative samples, which were also chosen by lottery out of 100 negative samples. Samples with inadequate volume or incomplete labelling, those with invalid or indefinite results by RT-PCR, and those with incomplete patient records were excluded. The flow chart of enrollment of samples is presented in Fig. 1.

### Data collection

#### Record review

The socio-demographic (age and gender) and clinical data were collected by reviewing the COVID-19 registration book for each participant including the number of days post-symptom onset, type of symptoms and chronic diseases using a data collection checklist. The following symptoms were recorded: cough, fever, back pain, sore throat, loss of smell, loss of taste, fatigue, and shortness of breath. Patients’ status for having chronic diseases such as hypertension, diabetes, tuberculosis, asthma, human immunodeficiency virus (HIV), chronic cardiac disease, and chronic kidney disease were also recorded.

### Laboratory analysis

#### Sample collection, transportation, and storage

The initial collection and storage of the nasopharyngeal samples utilised in the current investigation was done as follows. The collection of nasopharyngeal swabs from patients suspected of having SARS-CoV-2 infection was routinely done by trained healthcare professionals, following the proper infection control procedures and using the recommended personal protective equipment. The collected swabs were kept in a viral transport medium containing 3 ml fluid composed of gelatin and antimicrobial agents in a buffered salt solution. The specimens were transferred to the laboratory by maintaining a cold chain (2–4 °C). All samples were analyzed by using RT-PCR (Veri-Q PCR 316 assay, MiCo BioMed.Co., Ltd., Korea), and results were released for clinical management as soon as possible. All SARS-CoV-2-positive and 5% negative samples were stored at − 70 °C. For this performance evaluation, stored nasopharyngeal samples were re-tested using Veri-Q PCR 316 assay to confirm results.

### RT-PCR assay

#### SARS-COV-2 RNA extraction

Nucleic acid extraction was done by a trained laboratory technologist under biosafety level 2 (BSL-2) using the BIOER system (GenePure Pro fully automatic Nucleic Acid Purification System; Hangzhou Bioer Technology Co., Ltd., China), according to the manufacturer’s instructions. The extraction procedure took only 15 min to extract the SARS-CoV-2 ribonucleic acid (RNA) from a 300 µl nasopharyngeal specimen. In a single run, the system could be able to give us 32 extracts of the SARS-CoV-2 RNA. Then, the extract was transported to the room devoted to the master mix preparation and amplification.

#### SARS-COV-2 RNA detection

In the master mix preparation and amplification room, 63 μl of the reaction mix was prepared under the BSL-2 from 45 μl 2X One-Step RT-PCR master mix, 9 μl of COVID-19 primer and probe, and 9 μl internal positive control. Immediately after the preparation, a 7 μl of the mix was applied to the Eppendorf tubes each containing 3 μl of the SARS-CoV-2 RNA extract. A 10 μl of the reaction was applied to the lab chip by using a pipette and loaded into the RT-PCR for the amplification. The Veri-Q PCR 316 assay, designed for the qualitative detection of the open reading frame (ORF3a) and nucleocapsid (N) genes of SARS-CoV-2 RNA from nasopharyngeal swabs, was used to carry out the RT-PCR amplification. A positive control template and negative amplification control with nuclease-free water were included in each run. In the one-step reaction, the reverse transcription, which produces complementary deoxyribonucleic acid (cDNA), was done by heating the mix at 50 °C for 5 min. The cDNA was heated at 95 °C for 15 min (initial denaturation), followed by 45 cycles that each included denaturation at 95 °C for 8 s, annealing, and extension at 56 °C for 13 s. The RT-PCR includes 45-cycle amplifications, following the manufacturer’s instruction. The result is interpreted as positive when ORF3a and N genes, or only ORF3a gene, are amplified in fewer than 45 cycles. The viral load was expressed as a cycle threshold (CT) value and CT values < 40 were considered as positive. The analyzed samples, which showed an exponential fluorescence curve and a CT value ≥ 40 or no CT values, were considered negative.

### Rapid diagnostic tests

The Panbio COVID-19 antigen test device (Abbott Diagnostic GmbH, Jena, Germany) and the STANDARD Q COVID-19 antigen test (SD Biosensor, Korea) are an in vitro diagnostic rapid test that employs the lateral flow assay principle for the qualitative detection of SARS-CoV-2 antigen in nasopharyngeal swab specimens. The anti-SARS-CoV-2 antibody is conjugated with colour particles and is used as a detector for the SARS-CoV-2 antigen. The test procedures for both RDTs involve mixing using a vortex mixer for 10 s to disrupt thick mucus. For the Panbio test, 5 drops of samples are applied to the sample pad of the cassette, and results are read within 15–20 min of incubation. For the STANDARD Q test, 3 drops of samples are applied to the sample pad of the cassette and results are read within 15–30 min of incubation. A positive test result is indicated when control (C) and test lines are visible, and a negative result is when only control line (C) is visible. The test is invalid when the control line is invisible. Personnel, who perform RDTs and RT-PCR tests, were blinded to any demographic, clinical, and prior test results by assigning a sample code to replace any identifier. All the test procedures were done following each manufacturer’s instructions.

### Data analysis

Data were double-entered into Epidata version 4.6 and exported to SPSS version 25 (IBM Corp, Inc., New York, USA) for analysis. Frequencies and percentages were used for the description of the qualitative data. All continuous data were expressed as median and interquartile range (IQR). The sensitivity, specificity, positive predictive value (PPV), and negative predictive value (NPV) were calculated to determine the diagnostic performance of the RDTs, considering the RT-PCR as a reference method. Cohen’s Kappa index was computed to test the level of agreement. The confidence intervals (CI) were calculated using the GraphPad Prism version 9.5.1 for Windows (GraphPad Software, San Diego, California USA).

### Ethical clearance

The Institutional Review Board (IRB) of Hawassa University College of Medicine and Health Sciences evaluated and approved our study (Reference Number IRB/162/14), and informed consent was waived. We confirm that all methods were performed in accordance with the relevant guidelines and regulations. Code numbers were used in place of identifiers to ensure the confidentiality of collected information and blind the investigators to prior test results or participants’ demographic and clinical information.

## Results

### Participants’ demographic and clinical characteristics

A total of 200 stored nasopharyngeal samples (100 SARS-CoV-2 positives and 100 negatives), which were initially collected for routine available care, were analyzed in the present study from June 1 to July 30, 2022. The participants’ ages ranged from 1 to 85 years, with the median (IQR) age being 26 (13–38) years. More than half of the participants, 114 (57.0%), were males, and 86 (43.0%) were females. The majority of the participants, 163 (81.5%), were symptomatic as they showed at least one of the symptoms. Fever, 121 (60.5%), is the symptom most frequently reported in the patients included in the study, followed by cough, 90 (45.0%). The most frequently reported chronic disease among the participants was hypertension, 35 (17.5%), followed by diabetes mellitus, 27 (13.5%), and tuberculosis, 23 (11.5%) (Table 1).

**Table 1:** Demographic and clinical characteristics of study participants, Hawassa, June 2022.

Features	Category	RT-PCR positive	RT-PCR negative	Total
		n = 100	n = 100	n = 200
Demographic characteristics				
Sex	Male	54 (47.4%)	60 (52.6%)	114 (57.0%)
	Female	46 (53.5%)	40 (46.5%)	86 (43.0%)
Age (years)	< 18	29 (33.3%)	58 (66.7%)	87 (43.5%)
	≥ 18	71 (62.8%)	42 (37.2%)	113 (56.5%)
Clinical characteristics				
Any symptom	Yes	78 (47.9%)	85 (52.1%)	163 (81.5%)
	No	22 (59.5%)	15 (40.5%)	37 (18.5%)
Cough	Yes	52 (57.8%)	38 (42.2%)	90 (45.0%)
	No	48 (43.6%)	62 (56.4%)	110 (55.0%)
Fever	Yes	70 (57.9%)	51 (42.1%)	121 (60.5%)
	No	30 (38.0%)	49 (62.0%)	79 (39.5%)
Sore throat	Yes	13 (41.9%)	18 (58.1%)	31 (15.5%)
	No	87 (51.5%)	82 (48.5%)	169 (84.5%)
Shortness of breath	Yes	33 (49.3%)	34 (50.7%)	67 (33.5%)
	No	67 (50.4%)	66 (49.6%)	133 (66.5%)
Headache	Yes	40 (75.5%)	13 (24.5%)	53 (26.5%)
	No	60 (40.8%)	87 (59.2%)	147 (73.5%)
Loss of taste	Yes	8 (44.4%)	10 (55.6%)	18 (9.0%)
	No	92 (49.5%)	90 (50.5%)	182 (91.0%)
Loss of smell	Yes	8 (61.5%)	5 (38.5%)	13 (6.5%)
	No	92 (49.2%)	95 (50.8%)	187 (93.5%)
Fatigue	Yes	38 (67.9%)	18 (32.1%)	56 (28.0%)
	No	62 (43.1%)	82 (56.9%)	144 (72.0%)
Back pain	Yes	26 (78.8%)	7 (21.2%)	33 (16.5%)
	No	74 (44.3%)	93 (55.7%)	167 (83.5%)
Any chronic disease	Yes	44 (50.6%)	43 (49.4%)	87 (43.5%)
	No	56 (49.6%)	57 (50.4%)	113 (56.5%)
Hypertension	Yes	20 (57.1%)	15 (42.9%)	35 (17.5%)
	No	80 (48.5%)	85 (51.5%)	165 (82.5%)
Diabetes mellitus	Yes	10 (37.0%)	17 (63.0%)	27 (13.5%)
	No	90 (52.0%)	83 (48.0%)	173 (86.5%)
Chronic cardiac disease	Yes	3 (18.8%)	13 (81.2%)	16 (8.0%)
	No	97 (52.7%)	87 (47.3%)	184 (92.0%)
Chronic kidney disease	Yes	1 (12.5%)	7 (87.5%)	8 (4.0%)
	No	99 (51.6%)	93 (48.4%)	192 (96.0%)
Tuberculosis	Yes	10 (43.5%)	13 (56.5%)	23 (11.5%)
	No	90 (50.8%)	87 (49.2%)	177 (88.5%)
Asthma	Yes	8 (100%)	0 (0.00%)	8 (4.0%)
	No	92 (47.9%)	100 (52.1%)	192 (96.0%)
HIV	Yes	2 (40.0%)	3 (60.0%)	5 (2.5%)
	No	98 (50.3%)	97 (49.7%)	195 (97.5%)

HIV, human immunodeficiency virus; RT-PCR, real-time polymerase chain reaction.

### RDTs results in relation to RT-PCR

The diagnostic performance results of the RDTs compared with the RT-PCR are shown in Table 2. The Panbio COVID-19 antigen test detected the SARS-CoV-2 antigens in 89 samples; of which, 88 samples had also positive results by the reference method. Moreover, 99 negative results were concordant with the RT-PCR, yielding an accuracy of 93.5% for the detection of SARS-CoV-2 infection. Also, the STANDARD Q COVID-19 antigen test detected the SARS-CoV-2 antigens in 92 samples; of which, 91 samples had also positive results by the reference method. Moreover, 49 negative results out of 50 were concordant with the RT-PCR, yielding an accuracy of 93.3% for the detection of SARS-CoV-2 infection. Twelve and nine false negative test results were observed for the Panbio and the STANDARD Q, respectively, while only one false positive result was observed for both RDTs.

**Table 2:** Summary of the results of the Panbio and STANDARD Q COVID-19 antigen rapid test devices compared to the RT-PCR, Hawassa, June 2022.

	Reference method (RT-PCR)		
	Positive	Negative	Total
Panbio positive	88	1	89
Panbio negative	12	99	111
Total	100	100	200
Sensitivity (95% CI)	88.0% (79.9–93.6)		
Specificity (95%CI)	99.0% (94.5–99.9)		
Positive predictive value (95% CI)	98.9% (92.6–99.8)		
Negative predictive value (95% CI)	89.2% (82.9–93.4)		
Agreement (kappa; 95% CI)	87.0% (80.2–93.4)		

	Reference method (RT-PCR)		
	Positive	Negative	Total
STANDARD Q positive	91	1	92
STANDARD Q negative	9	49	58
Total	100	50	150
Sensitivity (95% CI)	91.0% (83.6–95.8)		
Specificity (95% CI)	98.0% (89.4–99.9)		
Positive predictive value (95% CI)	98.9% (92.9–99.8)		
Negative predictive value (95% CI)	84.5% (74.4–91.0)		
Agreement (kappa; 95% CI)	86.0% (77.0–94.2)		

CI, confidence interval; RT-PCR, real-time polymerase chain reaction.

### RDTs performance by age, clinical characteristics, and CT values

Table 3 shows the performance of the Ag-RDTs according to the presence of signs/symptoms, age, CT value, and presence or absence of chronic diseases. The higher performance of the test was observed for CT < 25, for which the sensitivity was 93.9% for the Panbio and 100% for STANDARD Q; and for age ≥ 18 years, the sensitivity was 98.6% for both RDTs. Regarding chronic disease, comparable sensitivities were observed.

**Table 3:** Diagnostic performance of the Panbio and STANDARD Q in different subgroups for RT-PCR positive participants, Hawassa, June 2022.

Overall	Ag-Test positive	Ag-Test negative	Sensitivity (95% CI)
CT value < 25			
Panbio (n = 49)	46	3	93.9% (83.1–98.7)
STANDARD Q (n = 49)	49	0	100% (92.8–100)
CT value ≥ 25			
Panbio (n = 51)	42	9	82.4% (69.1–91.6)
STANDARD Q (n = 51)	42	9	82.4% (69.1–91.6)
Age < 18 years			
Panbio (n = 29)	18	11	62.1% (42.3–79.3)
STANDARD Q (n = 29)	21	8	72.4% (52.8–87.3)
Age ≥ 18 years			
Panbio (n = 71)	70	1	98.6% (92.4–100)
STANDARD Q (n = 71)	70	1	98.6% (92.4–100)
Any symptom Yes			
Panbio (n = 78)	69	9	88.5% (79.2–94.6)
STANDARD Q (n = 78)	72	6	92.3% (84.0–97.1)
Any symptom No			
Panbio (n = 22)	19	3	86.4% (65.1–97.1)
STANDARD Q (n = 22)	19	3	86.4% (65.1–97.1)
Any chronic disease Yes			
Panbio (n = 44)	39	5	88.6% (75.4–96.2)
STANDARD Q (n = 44)	39	5	88.6% (75.4–96.2)
Any chronic disease No			
Panbio (n = 56)	49	7	87.5% (75.9–94.7)
STANDARD Q (n = 56)	52	4	92.9% (82.7–98.0)

Ag, antigen; CI, confidence interval; CT, cycle threshold; RDT, rapid diagnostic test.

For patients visiting within 3 days post symptom onset, the sensitivity of both RDTs was 96.3% (n = 77). For those presenting on or after 8 days post symptom onset, the sensitivity was 64.3% for STANDARD Q and 35.7% for the Panbio (Table 4).

**Table 4:** Performance evaluation of antigen rapid diagnostic tests against RT-PCR regarding days after symptom onset, Hawassa, June 2022.

Duration of symptom onset	Median days	Standard Q		Panbio	
1–3 days	2	Positive samples (n/N)	77/80	Positive samples (n/N)	77/80
		Sensitivity (95% CI)	96.3% (89.4–99.2)	Sensitivity (95% CI)	96.3% (89.4–99.2)
4–7 days	6	Positive samples (n/N)	5/6	Positive samples (n/N)	6/6
		Sensitivity (95% CI)	83.3% (35.9–99.6)	Sensitivity (95% CI)	100% (54.1–100)
≥ 8 days	11	Positive samples (n/N)	9/14	Positive samples (n/N)	5/14
		Sensitivity (95% CI)	64.3% (27.8–77.0)	Sensitivity (95% CI)	35.7% (12.7–64.8)

CI, confidence interval.

From the analysis of 100 samples from RT–PCR-confirmed positive individuals, the median (IQR) CT value for Panbio positive samples was 24.7 (19.6–26.7), whereas the median (IQR) CT value for false negative samples was 30.7 (24.9–34.2). The median (IQR) CT value for STANDARD Q positive samples was 24.6 (19.6–26.3) whereas, 34.0 (30.1–35.03) for false negative samples. Furthermore, the median (IQR) days post-symptom onset for Panbio and STANDARD Q false negative samples were 11.5 (2–14) and 7 (1–9.5), respectively (Table 5).

**Table 5:** The median and interquartile range of CT values and days post symptoms onset for true positive and false negative results, Hawassa, June 2022.

COVID-19 Ag-RDTs	RT-PCR (+)/Ag-RDT (+)	RT-PCR (+)/Ag-RDT (−)
	Median CT value [IQR]	
Panbio	24.7 [19.6–26.7]	30.7 [24.9–34.2]
STANDARD Q	24.6 [19.6–26.3]	34.0 [30.1–35.03]
Median days post symptom onset [IQR]		
Panbio	3 [2–3]	11.5 [2–14]
STANDARD Q	3 [2–3]	7 [1–9.5]

Ag-RDT, antigen rapid diagnostic test; CT, cycle threshold; IQR, interquartile range; RT-PCR, real-time polymerase chain reaction.

## Discussion

The pandemic response depends heavily on improving diagnostic access to SARS-CoV-2 detection in COVID-19-suspected people. Thus, developing accurate methods for the prompt detection of SARS-CoV-2 is crucial. We evaluated the performance of the Panbio and the STANDARD Q COVID-19 antigen tests relative to the RT-PCR for the diagnosis of SARS-CoV-2. It is due to the need to find a reliable point-of-care test, which could effectively reduce the burden of specimen referral logistics and contribute to improved diagnostic coverage in resource-limited areas. The respective sensitivity and specificity were 88.0% and 99.0% for the Panbio and 91.0% and 98.0% for the STANDARD Q. The positive predictive value for both RDTs was 98.9%, while the negative predictive values were 89.2% and 84.5% for the Panbio and STANDARD Q, respectively.

The performance demonstrated in our study was in line with the previous studies^10,11^. However, our findings indicated lower sensitivities compared to reports from Thailand (98.3%)^7^ and Korea (94.9%)^12^ for the STANDARD Q; and from Ethiopia (95%) for Panbio^13^. Possible explanations for the relatively lower sensitivities in our study might be associated with the lower viral loads in our study participants. On the other hand, our results were higher than the sensitivities reported for the STANDARD Q (17.5–70.6%)^6,14,15^, and for the Panbio (41.3–81.0%)^11,16,17^. This might be due to the factors that influence the performance of Ag-RDTs, such as the type and quality of the specimen, collecting and processing techniques, and the transportation and storage conditions.

Regarding viral load, both RDTs were shown to have higher sensitivities in samples with lower cycle thresholds (CT value < 25) compared to those with higher ones (CT value ≥ 25). This finding was in agreement with results reported in the Netherlands^16^, Korea^12^, and Spain^18^. Similar results have been found by other research in samples with low CT values due to high virus loads^11,13,19,20^. This might suggest that an elevated viral load, which is connected to viral infectivity, is indicated by a positive antigen test^21^.

Moreover, a higher sensitivity of 98.6% (95% CI 92.4–100%) was shown for both Panbio and STANDARD Q among participants aged 18 years and above compared to those aged less than 18 years, as reported by other studies^20,22^. However, no age-related difference was observed in SARS-CoV-2 viral loads between children and adults^11^. Although the onset of the symptom is harder to identify in children than in adults, which may influence comparison, the proportion of individuals having a CT value of ˂ 25 was higher in the 18 years and older group compared to the under-18 age group (53.5% versus 37.9%, respectively).

We also provided data showing that the likelihood of positive Ag-RDT results matched with the early onset of symptoms, consistent with the findings of other studies^10,15,19,23^. It was observed that the sensitivities of the RDTs were higher in participants with ≤ 7 days post-symptom onset compared to those with the onset > 7 days, which might be due to reduced antigen concentrations in samples from participants with late-stage symptoms. Moreover, participants with symptoms > 7 days were more likely to have lower viral loads in nasopharyngeal swabs than those who were tested at an early stage after the onset of symptoms.

Our findings of high specificities of the RDTs were in line with other similar research results (97.6–100%)^4,11,20^. It might imply that rapid antigen tests have a very low likelihood of producing false positive results and that a subject who receives a negative test is very likely to be negative for SARS-CoV-2. However, a report of a lower specificity for STANDARD Q (81%)^7^ compared to our result, may be caused by cross-reacting antibodies from prior infections or the test’s performance being impacted by the environmental conditions under which the test was performed.

Diagnostic tests with a high positive predictive value would be essential to assist efforts in reducing the risk of transmission of infection^9^. Both of the Ag-RDTs used in this study had positive predictive values of 98.9%, indicating that individuals who test positive via the Ag-RDTs are very likely to be infected with the SARS-CoV-2 virus. Thus, the use of Ag-RDTs might make diagnostics more accessible, especially in countries with limited resources, and make it easier to undertake mass screening for COVID-19.

To our knowledge, this is the first study evaluating the Panbio and STANDARD Q COVID-19 antigen tests against RT-PCR, providing insight into the performance characteristics of these kits and enabling evidence-based decisions for their potential use in large-scale COVID-19 testing in countries with limited laboratory capacity. However, our study has some limitations. Due to a shortage of the STANDARD Q test kits, we tested a reduced number of negative samples. It would have also been desirable if we had included prospective clinical data. Further, although there were other commercially available RDTs, we only evaluated the performance of two RDTs.

## Conclusion

The Panbio and STANDARD Q COVID-19 antigen tests showed excellent performance in detecting the SARS-CoV-2 infected cases and fulfilled the World Health Organization recommendations (≥ 80% sensitivity and ≥ 97% specificity compared to the RT-PCR) for the use of these tests. However, a further investigation with a larger sample size on fresh nasopharyngeal specimens collected at the point of care would be essential to confirm the observed diagnostic performance and integrate them into clinical guidelines.

## Data Availability

The datasets generated and/or analyzed during the current study are available from the corresponding author, upon reasonable request and with the Institutional Review Board of the Hawassa University College of Medicine and Health Sciences.
